# The application of observational data in translational medicine: analyzing tobacco-use behaviors of adolescents

**DOI:** 10.1186/1479-5876-10-89

**Published:** 2012-05-14

**Authors:** Valeria Siciliano, Annalisa Pitino, Mercedes Gori, Olivia Curzio, Loredana Fortunato, Michael Liebman, Sabrina Molinaro

**Affiliations:** 1 Clinical Physiology Institute, National Research Council, Pisa, Italy; 2 Strategic Medicine, Inc, 231 Deepdale Drive, Kennett Square, PA 19348, USA

**Keywords:** Translational Medicine, Observational Study, Tobacco Use, Adolescent Behaviour, Government Policy

## Abstract

**Background:**

Translational Medicine focuses on “bench to bedside”, converting experimental results into clinical use. The “bedside to bench” transition remains challenging, requiring clinicians to define true clinical need for laboratory study. In this study, we show how observational data (an eleven-year data survey program on adolescent smoking behaviours), can identify knowledge gaps and research questions leading directly to clinical implementation and improved health care. We studied gender-specific trends (2000–2010) in Italian students to evaluate the specific impact of various anti-smoking programs, including evaluation of perceptions of access to cigarettes and health risk.

**Methods:**

The study used, ESPAD-Italia® (European School Survey Project on Alcohol and other Drugs), is a nationally representative sample of high-school students. The permutation test for joinpoint regression was used to calculate the annual percent change in smoking. Changes in smoking habits by age, perceived availability and risk over a 11-year period were tested using a gender-specific logistic model and a multinomial model.

**Results:**

Gender-stratified analysis showed 1) decrease of lifetime prevalence, then stabilization (both genders); 2) decrease in last month and occasional use (both genders); 3) reduction of moderate use (females); 4) no significant change in moderate use (males) and in heavy use (both genders). Perceived availability positively associates with prevalence, while perceived risk negatively associates, but interact with different effects depending on smoking patterns. In addition, government implementation of public policies concerning access to tobacco products in this age group during this period presented a unique background to examine their specific impact on behaviours.

**Conclusion:**

Large observational databases are a rich resource in support of translational research. From these observations, key clinically relevant issues can be identified and form the basis for further clinical studies. The ability to identify patterns of behaviour and gaps in available data translates into new experiments, but also impacts development of public policy and reveals patterns of clinical reality. The observed global decrease in use is countered by stabilization in number of heavy smokers. Increased cigarette cost has not reduced use. While perceived risk of smoking may prevent initial experimentation, how government policies impact the perception of risk is not easily quantifiable.

## Introduction

The challenges and opportunities for translational medicine (TM) were well described in an editorial in 2003 [1], where the difference between the “bench to bedside” and “bedside to bench” paradigms were described. As noted, the emphasis on TM has been on moving research results from the experimental domain into the clinic, but the success of this approach has been limited as many such experiments are driven by conventional, hypothesis-driven basic research and not a direct association with true clinical need [2]. There are at least two approaches to drive the bedside to bench paradigm that could increase the potential that research outcome will have direct clinical application: 1) apply knowledge engineering approaches to identify concerns, gaps and critical need from the clinician, possibly using a natural language interface [3]; and 2) appropriately data-mine and utilize the ex vivo data that exist in well-designed observational studies. This report focuses on the second option and applies it to the analysis of smoking behaviours in adolescents over an 11 year period. Smoking directly impacts an individual’s health (and quality of life) and indirectly impacts economic factors because of both the increased healthcare costs and lost time at work. Globally, public awareness and anti-smoking programs have reduced the use of tobacco [4-8].

Smoking behaviours represent a process of evolution, from initial experimentation to controlled use and finally addictive behaviour. Recent studies describe changes in smoking habits related to changes in tobacco control policies, in order to analyze which programs could prevent the initiation of smoking as well assist in ending of this addictive behaviour [8].

Our analysis further examines the impact of specific programs and recognizes the difference between experimentation and its progressive conversion to long-term smoking habits (occasional, moderate or heavy use), also including evaluation of self-perception of access to cigarettes and health risk, especially in years when the programs have been applied.

It is critical to understand early behaviours of smokers, particularly adolescents. In most cases, people begin to smoke during adolescence with nearly 25% smoking their first cigarette before age 10 [9]. Unfortunately, smoking habits among adolescents change over time, and it is a public health imperative to monitor smoking prevalence; this is critical to specify the problem, establish countermeasures and evaluate public health efforts to reduce smoking prevalence [10-12]. Young smokers prevalence fluctuates and it is difficult to define a unique population dynamic that describes smoking habits. Overall, from 1995 to 2007, the quadri-annual ESPAD, European School Survey Project on Alcohol and other Drugs (20 countries), showed a significant decline (North-West Europe), a significant increase/stabilization over the first years followed by a decrease (Central-South Europe), an increase or a stagnation (East Europe) [6].

Many variables have been shown to influence adolescent smoking behaviour, including: gender, education, parental and peers smoking habits, access to disposable income, socio-economic status, availability of cigarettes, perceived risk and environmental variables like tobacco control policies [13-19].

**Figure 1:**
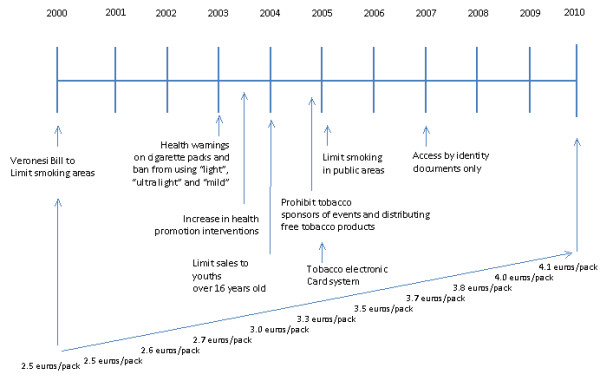
Timeline of anti-smoking intervention programs in Italy during period of ESPAD data collection.

Simple programs to address all such factors are difficult to define. In alignment with European directives, during the last 20 years in Italy, tobacco control interventions have been planned on the basis that perception of high risk and/or decreased availability have a “protective effect” on smoking behaviour. Attention to health consequences from smoking further is evident in awareness campaigns on the risk of smoking carried out since 2000. The timeline of intervention programs in Italy is shown in Figure 1.

It is of interest to analyse whether anti-tobacco programmes could influence adolescents: by preventing experimentation with tobacco, or reducing the chance that an experimenter would become an established smoker. Raising the price of tobacco and prohibiting smoking in public places, are two policies which have had the greatest impact on smoking rates [18]. Adolescent public education programs and limits on retail sales can have a large impact when combined with other policies [18,19]. Hypothetically, these programs could reduce adolescent use through: 1) smoking cessation (result in reduced prevalence in last month), 2) reducing smoking levels (resulting in conversion of heavy or moderate smokers to occasional) and 3) prevent smoking altogether (i.e. reduced prevalence in lifetime). For this reason, it is important to understand which tobacco control policies could influence the perceived risk and/or the perception of access to cigarettes, how this would affect smoking habits, and whether this may be gender specific.

In particular, risk perception results from a complex set of issues that combine elements of belief with a subjective valuation of specific outcome. A large literature documents gender and age differences in risk perception. Gender-based studies usually show that women are more sensitive to the perception of risk than men. However, using Gustafson’s words: “women and men perceive the same risk differently, they may perceive different risk, and they may attach different meanings to what appear to be ‘the same’ risks” [20]. Dividing potential risky behaviours in financial, health/safety, recreational, ethical and social decisions domains, it is shown that males perceive less risk and a greater likelihood of engaging in risky behaviour in the first four [21,22]. Based on a gender perspective, the interpretation of the differences in risk perception may refer to gender structures, segregation and hierarchy, and be moderated by other structural factors, such as economic class, or individual factors [20].

Concerning adolescents’ risk perception, youths tend to underestimate risk, especially associated with tobacco use, because of the time interval between initiation of smoking and impact on health and also because of positive reinforcement from social interactions. Stjerna et al. suggest that, although well informed about health risk, smoking is generally considered acceptable during teenage years, resulting in dangerous consequences only for adult smokers [17].

The perceived availability of a specific substance, although related to a self-perception, is recognized to be affected by an individual’s environment, and can represent a ”general” indicator of the accessibility of the substance. Environmental factors as in particular the influence of peer group (perceived use among friend and siblings) and perceived availability seem moreover more important than parental control and family structure in the “prediction” of use [23,24].

The current study has uniquely analyzed changes in the early smoking habits of Italian adolescents (330,000 students) from 2000 to 2010. We present this detailed analysis as the basis for examining the specific impact of various anti-smoking programs to evaluate their differences in altering experimentation and long-term smoking behaviours, under the hypothesis that there exists a strict association among each intervention program, perceived availability and perceived health risk, and smoking habits.

## Methods

### Sampling and data collection

This study uses data from school surveys conducted annually since 2000, which provide a continuous record of drug, alcohol and tobacco use among Italian students*.* Questionnaires are self-administered (March-April) to a representative sample of high school students, aged 15–19 years according to the ESPAD methodology. The students have been adequately informed and data collection was performed using anonymous questionnaires completed in the classroom; participation was completely voluntary [6,25]. Sample characteristics are reported in Table 1 (note: variation in sample size results from levels of funding support on an annual basis, but response rates average is around 93%).

**Table 1:** Sample characteristics. Years 2000-2010

**Characteristic**	**2000**	**2001**	**2002**	**2003**	**2004**	**2005**	**2006**	**2007**	**2008**	**2009**	**2010**
N	22,418	22,257	15,752	25,299	32,372	41,365	38,748	40,407	38,681	32,461	25,555
Age (mean±SD)	17.1 ± 1.5	17.1 ± 1.4	17.2 ± 1.6	17.1 ± 1.6	17.1 ± 1.6	17.1 ± 1.6	17.1 ± 1.6	17.1 ± 1.6	17.2 ± 1.6	17.1 ± 1.6	17.1 ± 1.4
Gender (male)	47.3%	45.0%	45.5%	45.5%	48.1%	48.1%	48.9%	49.7%	49.0%	49.2%	47.9%
Response rate*	100.0%	87.1%	98.6%	94.9%	96.1%	94.1%	88.9%	92.4%	85.8%	89.2%	86.2%

* Response rate of schools participating in the survey; SD = Standard Deviation.

### Instruments and measures

The questionnaire used, consisting of core questions about legal and illegal activities, was essentially identical throughout the entire period of this study [6].

The questions focused on smoking experience, smoking frequency, perceived risk and perceived availability to cigarettes. Cigarette use variables were examined over an individual’s lifetime and last 30-days prevalence.

1. “*On how many occasions (if any) during your lifetime have you smoked cigarettes*?”:

“never, once or twice, 3–5 times, 6–9 times, 10–19 times, 20–39 times and 40 times or more” and

2. “*How frequently have you smoked cigarettes during the last 30-days*?”:

“not at all, less than 1 cigarette/week, less than 1 cigarette/day, 1–5 cigarettes/day, 6–10 cigarettes/day, 11–20 cigarettes/day, more than 20 cigarettes/day”.

– Referring to the last 30-days, people were classified in:

– occasional smokers (less than one cigarette/day)

– light/moderate (1–10 cigarettes/day),

– heavy (11 or more cigarettes/day)

We define “heavy smokers” (11 or more cigarettes/day) like other authors studying adolescents [26,27]. However, we observe the prevalence of adolescents that have smoked 20 or more cigarettes/day varies from 1% (year 2001) to 2.8% (year 2004): this limited variation prevented a separate analysis. We have also considered smokers of less than one cigarette/day separately from light smokers, because it is extremely relevant to analyze the conversion from occasional to daily smoker (moderate or heavy).

Respondents were also asked about their perceived risk from smoking and their perceived cigarettes availability.

3) “*How much do you think PEOPLE RISK harming themselves (physically or in other ways) if they smoke one or more packs of cigarettes per day?”*

Perceived risk was separated as great risk versus other responses (moderate risk; slight risk; no risk; don’t know) [6].

4) “*How difficult do you think it would be for you to get cigarettes if you wanted?*.

Perceived availability was defined as very easy and fairly easy versus other responses (fairly difficult; very difficult; impossible; don’t know) [23,28].

To study the interaction between perceived availability and perceived risk we created a composite parameter, resulting in four levels: 0 = “not perceived risk and no availability”; 1 = “perceived risk but no availability”; 2 = “not perceived risk but perceived availability”; 3 = “perceived risk and availability”. In this new parameter, “no perceived risk” → “slight/no risk” and “no availability” → “fairly difficult, very difficult, impossible”.

The development and use of such composite parameters enables more flexible analysis to be performed using data that is more subjective than objective in nature. The analysis actually involves the combination of both subjective and objective data, e.g. specific smoking patterns and behaviours. The data collected in an observational study present additional challenges of complexity over studies that are designed to test a specific hypothesis, as in most clinical studies. The methods used to analyse observational study data must deal with multiple interacting observations that are not observed independently. In this particular observational study, during the period of its data collection, multiple changes in governmental policy were effected, thus impacting the potential to fully isolate the impact of any one policy, particularly given the potential length of time to observe a specific response. It is interesting to note, however, that those policies which were implemented prior to 2004 focused on enhanced communication of health risk while those in 2005 and beyond more specifically attempted to directly limit access to cigarettes in this population.

### Statistical analysis

This study is fundamentally observational therefore, no attempt was made to link specific policies with individual perceptions and behaviours during the data collection. Therefore, this analysis will look at the ESPAD-Italia® data from three perspectives in order to provide greater confidence in the results reported. A prevalence trend analysis (including differences between genders) was followed by logistic regression analysis (among the lifetime and last month use) and multinomial regression analysis (among last month users), in order to understand in which way the perceptions of risk and of availability affect different smoking typologies [29]. Because gender differences in perception have been reported in literature, the analysis is further stratified by gender.

Reported prevalence was weighted using the Italian school population average from 2005 to 2009 (only years available). At the basic level, differences in proportions by genders for each year were evaluated using chi-square tests.

After estimating smoking prevalence in each age-gender group (for each period), we used the permutation test for joinpoint regression to detect significant Annual Percent Change (APC) in prevalence (Joinpoint Regression Program 3.0) [30]. A maximum of two joinpoints and three line segments were allowed. We fitted the models on the log scale, using a weighted least square criteria, where the weight was the standard error of the observed prevalence. The slope of each continuous linear phase is interpreted as the period percent change in prevalence [31]. A gender-specific permutation test for joinpoint regression was performed for 1) perceived availability, 2) perceived risk, 3) prevalence of lifetime use, 4) last 30-days use, 5) occasional smokers, 6) moderate smokers and 7) heavy smokers. The test for pair-wise differences by-group was performed to evaluate “parallelism” in trends by gender.

Two gender-specific logistic regression models, age-adjusted, for both perceived risk and perceived availability were performed.

Then, a gender-specific logistic regression was computed for lifetime and last month use, as to make individual level adjustments for perceived availability and smoking risk together. In addition to age, in fact, the interaction between perceived risk and availability was added. A gender-specific multinomial regression model was fitted for the last month users, focusing only on smokers (moderate and heavy) and using as base category the “occasional smokers”, in order to understand the specific role of risk perception and the availability on the odds to be a smoker. Also in these models, age and the interaction between perceived risk and availability were added.

Two more variables were added to all logistic and multinomial models to assess trends. Firstly, a yearly sequential variable from 2000 to 2005 was included. This was used to measure long-term trend in prevalence before joinpoint year in perceived availability. Secondly, a yearly sequential variable from 2005 to 2010 was also included to capture trends that were limited to the post joinpoint year. We used the joinpoint year that detected the change in perceived availability as the joinpoint year for the perceived risk; this last one did not show considerable difference in the trend of prevalence use and it did not further explain the observations. Furthermore, the decrease in perceived availability was coincident with the implementation (in 2005) of policies that restricted access as noted above. The survey year was treated as a continuous (ordered categorical) variable.

The overall statistical significance level of the 2-sided analysis was p < 0.05. All regression models were performed using Stata v.10.1.

## Results

**Figure 2:**
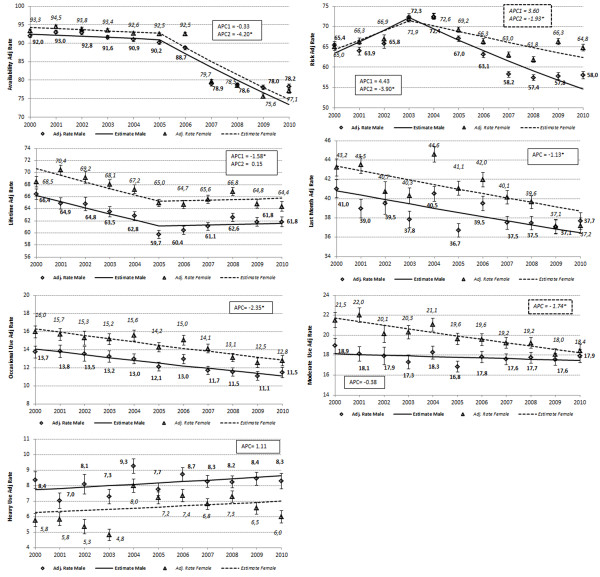
**Age-Adjusted Availability,Risk Perception and Smoking Prevalence in Italian students, from 2000 to 2009 (Joinpoint Trend).** 1) perceived availability, 2) perceived risk, 3) prevalence of lifetime use, 4) last 30-days use, 5) occasional smokers, 6) moderate smokers, 7) heavy smokers. * Indicates a significant Annual Percent Change (APC).

Figure 2 shows prevalence rates of cigarette use, perceived risk and perceived availability for both genders, adjusted by age. An adjusted rate is a weighted average of the age-gender specific crude rates, where the weights are the proportions of students (age/genders groups) in the standard population. Comparing adjusted rates reduces any potential confounding effect. Since using adjusted and crude rates reveals no differences, this implies that demographic differences are not significant. Globally, there is an evident gap between genders concerning smoking patterns and perception of cigarette availability and risk. With few exceptions, females show higher prevalence related to lifetime smoking behaviour, last month use, in occasional and moderate smokers, while males show higher prevalence within heavy smoking category.

Observation: from 2000 to 2010, both genders present a global decrease of perceived risk and availability of cigarettes. Prevalence of risk perception for females is higher after 2005 (no significant differences until 2004) while the perception of availability is lower for males until 2007 (P < 0.01). For further study: although post 2005 policies focused on limiting access, only, females became more sensitive to the potential risk of smoking during this time period, well beyond males. Is this a result of interaction between risk perception and reduced availability and how it is interpreted by females or are there other external factors that may have heightened their perception of risk?

The gender-stratified joinpoint analysis, confirmed 1) significant Annual Percent Change (APC) decrease of lifetime prevalence, then stabilization in both genders (in parallel); 2) decrease in last month use and occasional use in both genders (in parallel); 3) reduction of moderate use among females and stabilization of moderate use prevalence among males (in parallel) and 4) stabilization of heavy use in both genders (in parallel) (Figure 2).

Observation: concerning lifetime prevalence, results show that 2000 to 2005 lifetime prevalence dropped at an annual rate of 1.6%, whilst after 2005 there was no significant change. For further study: since policies implemented in this period specifically addressed issues of health risk from smoking, only, is this change due to increased perception of risk but confounded by smokers who established channels for access to cigarettes which were not impacted by the subsequent policies that attempted to limit access?

Observation: concerning smoking habits in the last month, the APC was −1.13% for both genders. The occasional use in last month decreased 2.4% per year for both genders, while moderate cigarette use in the last month decreased 1.7% per year only among females (Figure 2). The joinpoint analysis for perceived smoking risk and perceived availability shows a significant decrease in 2003 and 2005 respectively. For further study: the change in moderate use by females, only, appears to be an aggregate of the sensitivity to increased risk and reduced access throughout the period with the changes after 2005 revealing that the increased perception of risk in females may have had a more significant impact on moderate female smokers. By contrast moderate male smokers appear to be insensitive to increases in risk perception or limiting access. Does this suggest that the path from occasional to moderate to heavy smoking operates differently in males than in females? Would different programs targeting the genders more specifically be able to produce more similar outcomes in the two groups?

Observation: Concerning perceived risk prevalence dropped from 2003 at an annual rate of 3.9% among males and 1.9% among females, a not significant increase in prevalence before 2003 is shown. About perceived cigarettes availability, the joinpoint was in 2005 with a not significant reduction in the previous (2000–2005: APC −0.3%) and significant in the following (2005–2010: APC −4.2%) period. For further study: the programs that were implemented to reduce access to cigarettes, starting in 2005, appear to have produced significantly different results in females versus males, not necessarily in the perception of availability, alone, but perhaps in the overall attitude towards the combination of health risk and availability. Do the interaction of these two approaches operate differently across the genders or are there other external factors that might be partially responsible as well for these differences?

Logistic models were applied to analyze trends, age-adjusting, for perceived availability and perceived risk, by genders. Table 2 shows the Odds Ratios (OR) and Confidence Intervals (CI).

**Table 2:** Logistic model for perceived risk and availability among males and females

**High Risk Perceived**				
	**Males (M)**		**Females (F)**	
Parameter	OR (CI 0.95)	p-value	OR (CI 0.95)	p-value
Trend 2000–2005 (one year)	1.04 (1.03;1.05)	***	1.04 (1.03;1.05)	***
Trend 2005–2010 (one year)	0.89 (0.89;0.90)	***	0.94 (0.93;0.94)	***
Age (one year)	1.07 (1.06;1.07)	***	1.10 (1.09;1.10)	***
**High Availability**				
Parameter	OR (CI 0.95)	p-value	OR (CI 0.95)	p-value
Trend 2000–2005 (one year)	0.94 (0.92;0.95)	***	0.97 (0.96;0.98)	***
Trend 2005–2010 (one year)	0.81 (0.81;0.82)	***	0.75 (0.74;0.76)	***
Age (one year)	1.46 (1.44;1.47)	***	1.41 (1.40;1.43)	***

Odds Ratio (OR), Confidential Interval (CI).

*p < 0.05; **p < 0.01; ***p < 0.001.

Observation: the perceived risk of smoking and the perceived availability increased with age in both genders. For both, we observed an increase in perceived risk during the first period, and a decrease in the period after. A decrease was observed for the perceived availability in the first period, and a higher decrease in the second one. For further study: it is clear that the increased sensitivity with age towards perceived risk and perceived availability may be associated with increased maturity and experience in the adolescents being studied, but does this suggest that there could be specific modifications to the policies and programs being implemented that would address this change, e.g. address the realization by youth that the health impact is not necessarily something that they can escape just because they do not see the specific effects during their adolescent years?

Logistic models were applied to evaluate differences between genders in individuals characteristics on lifetime and last month use. Table 3 shows the Odds Ratios (OR) and Confidence Intervals (CI).

**Table 3:** Logistic model for prevalence use in lifetime and last month among males and females

**Lifetime use**				
	**Males (M)**		**Females (F)**	
Parameter	OR (CI 0.95)	p-value	OR (CI 0.95)	p-value
Trend 2000–2005 (one year)	0.95 (0.94;0.96)	***	0.96 (0.95;0.96)	***
Trend 2005–2010 (one year)	1.04 (1.03;1.04)	***	1.05 (1.04;1.06)	***
Age (one year)	1.30 (1.29;1.31)	***	1.27 (1.26;1.28)	***
(Risk & No Availability) vs (No Risk & No Availability)	0.59 (0.56;0.63)	***	0.63 (0.60;0.67)	***
(No Risk & Availability) vs (No Risk & No Availability)	3.59 (3.42;3.76)	***	4.71 (4.47;4.97)	***
(Risk & Availability) vs (No Risk & No Availability)	2.44 (2.33;2.55)	***	2.93 (2.79;3.08)	***
**Last month use**				
Parameter	OR (CI 0.95)	p-value	OR (CI 0.95)	p-value
Trend 2000–2005 (one year)	0.99 (0.98;1.00)	n.s.	1.00 (0.99;1.01)	n.s.
Trend 2005–2010 (one year)	1.00 (0.99;1.01)	n.s.	1.00 (0.99;1.00)	n.s.
Age (one year)	1.30 (1.29;1.31)	***	1.17 (1.16;1.18)	***
(Risk & No Availability) vs (No Risk & No Availability)	0.51 (0.47;0.55)	***	0.53 (0.48;0.57)	***
(No Risk & Availability) vs (No Risk & No Availability)	3.55 (3.34;3.76)	***	4.82 (4.51;5.15)	***
(Risk & Availability) vs (No Risk & No Availability)	2.31 (2.18;2.44)	***	2.98 (2.80;3.18)	***

Odds Ratio (OR), Confidential Interval (CI).

*p < 0.05; **p < 0.01; ***p < 0.001.

Observation: for lifetime use, in both genders, the analysis shows a significant decrease before 2005 and an increase after. The prevalence increases significantly with age in both genders. Students with perception of smoking risk and no availability of cigarettes are more “protected” against smoking (no significant difference in odds ratio between genders: OR(M) = 0.59; OR(F) = 0.63). The odds to smoke cigarettes increases gradually if students perceive also availability (higher significant association for females). Not to perceive risk but to perceive availability is more positively associated with smoking prevalence compared to the other categories. For further study: it is not surprising that students may have a sense of risk but not a full commitment or realization of its true impact from smoking but the added barrier of reduced access is sufficient to curtail their efforts. Does the path of smoking behavior from experimental to occasional use to moderate and then heavy use change if this barrier is high in the beginning, i.e. is this pattern of progression different now (since 2005) in time or degree than it was during the 2000–2004 period?

Observation: for the last month use, in both genders Table 3 shows no significant trend in both periods. The prevalence increases with age, and students in the category “perceived risk but no availability” are more “protected” (no significant difference in odds ratio between genders, but lower than in lifetime use). In addition, to perceive no risk but perceive availability is more positively associated with last month use (non significant differences with the odds ratio found in lifetime use).

Table 4 shows the Relative Risk Ratio (RRR) and Confidence Intervals (CI). For further study: this pattern appears similar to the moderate smokers’ one in the case of females. Does this suggest that there are significant differences in the access routes to cigarettes for females than for males and that may be an important element to consider for developing future policies to limit access overall?

**Table 4:** Multinomial regression model for moderate and heavy smokers referring to occasional ones among males and females

	**Males (M)**			
	**Moderate**		**Heavy**	
Parameter	RRR (CI 0.95)	p-value	RRR (CI 0.95)	p-value
Trend 2000–2005 (one year)	1.01 (0.99;1.02)	n.s.	1.05 (1.03;1.07)	***
Trend 2005–2010 (one year)	1.02 (1.01;1.03)	**	1.00 (0.98;1.01)	n.s.
Age (one year)	1.23 (1.21;1.24)	***	1.45 (1.42;1.48)	***
(Risk & No Availability) vs (No Risk & No Availability)	0.91 (0.77;1.08)	n.s.	0.71 (0.57;0.88)	**
(No Risk & Availability) vs (No Risk & No Availability)	1.62 (1.44;1.82)	***	1.50 (1.30;1.72)	***
(Risk & Availability) vs (No Risk & No Availability)	1.37 (1.22;1.53)	***	0.88 (0.77;1.01)	n.s
	**Females (F)**			
Parameter	RRR (CI 0.95)	p-value	RRR (CI 0.95)	p-value
Trend 2000–2005 (one year)	1.00 (0.98;1.01)	n.s.	1.09 (1.07;1.11)	***
Trend 2005–2010 (one year)	1.01 (1.00;1.02)	*	0.99 (0.97;1.00)	n.s
Age (one year)	1.27 (1.26;1.29)	***	1.42 (1.39;1.44)	***
(Risk & No Availability) vs (No Risk & No Availability)	0.77 (0.65;0.91)	***	0.47 (0.36;0.60)	***
(No Risk & Availability) vs (No Risk & No Availability)	1.92 (1.69;2.19)	***	1.84 (1.55;2.19)	***
(Risk & Availability) vs (No Risk & No Availability)	1.51 (1.33;1.72)	***	1.03 (0.86;1.22)	n.s.

Relative Risk Ratio (RRR), Confidential Interval (CI).

*p < 0.05; **p < 0.01; ***p < 0.001.

In order to analyse the last month users, we performed multinomial regression models. Observation: for both genders, age-adjusting, and controlling for interaction between perceived availability and risk, the trend in smoking prevalence over the first time period increased significantly for heavy smokers and is steady for the moderate ones; this implies a decrease of occasional smokers. In the second period, the trend is steady for the heavy smokers, increases for moderate ones and, accordingly, decreases for occasional smokers. A positive association was found with students' age, higher for heavy smokers, in both genders. For further study: anti-smoking intervention policies are mainly focused on prevention that appears to be mostly successful amongst occasional smokers. It is clear that heavy smokers exhibit reduced susceptibility to outside influences in determining their smoking habits. Does this persist when one analyses the time of progression from occasional smoking to moderate to heavy smoking, i.e. are there differences in behaviors and response to external policies in those heavy smokers who progress rapidly versus those who evolve over a longer time course?

*Moderate users*:

Observation: The category of students that “perceive no risk but perceive availability” is positively associated with cigarette use, in both genders. The odds for males to be a moderate user is not depending by risk (RRR(M) = 0.91; p > 0.05), but the odds increases in addition to perceive availability. For females, instead we found a more “protective” effect of perceived risk (RRR(F) = 0.77; p < 0.001) and higher association with perceived availability. For further study: does this pattern of experimentation with potentially addictive behaviors for females extend beyond cigarette smoking, e.g. alcohol or drug abuse, food addiction, etc? Would this suggest that programs need to be tailored differently to focus on the gender differences in perception and response?

*Heavy users*:

Observation: for both genders, “perceived no risk but availability” is more positively associated with heavy use. In addition, we observe that “to perceive risk and no availability” decreases the odds to be a heavy smoker (RRR(M) = 0.71; RRR(F) = 0.47)”, with a higher “protective” effect of risk, so there is no significant difference with “perceived risk and availability” (RRR(M) = 0.88; RRR(F) = 1.03; p > 0.05). For further study: the behaviour of heavy smokers appears to involve other underlying physiological responses that may counter social behaviors and need to be incorporated into programs that focus on this group. There is a need for tobacco control interventions that are specific to heavy smokers that can take into account socio-demographic and also neuro-psychological characteristics. Are these behaviors similar in other addictive substance or behaviour classes, e.g. alcohol or drug abuse, food addiction?

## Discussion

The general decrease of lifetime and last month cigarette use is in line with trends evidenced in non-Italian youths [6,7]. Joinpoint analysis suggests that specific policies could influence the perceptions of access to cigarettes and health risks, with greatest impact in years in which entered into force (Figure 2). Important interventions to restrict youth access occurred between 2004 and 2005, and to awaken public opinion to smoking risk in 2003 and 2004 (Figure 1).

In particular, policies directed towards limiting availability to cigarettes seem to affect perceived availability: we observe a steady decrease from 2000 to 2005, significant after 2005 and even greater after 2007 (access to cigarette machines requires an identity card). On this purpose it is important to underline that in 2005 enter into force the policies to control access, strongly required by the Framework Convention on Tobacco Control (FCTC 2003), limiting access to smoking (limit sales to >16 years old, limit smoking in public areas, tobacco electronic card system), as reducing advertisement and distributing free tobacco products during events (i.e. sporting events) (Figure 1). These policies are consistent with the literature that suggesting that a significant portion of youth experimentation can be attributed to promotional activities around tobacco [19], and in agreement with the general consensus that reducing sales to youth and/or increasing price can deter youths smoking. However other studies show that reduced sales to minors do not produce changes in adolescents’ perceptions of access or smoking habits, as youths obtain cigarettes from various sources (i.e. friends and parents) [19,32], such as adolescent smokers reduced daily cigarette consumption as prices rose, but compensated in other ways, e.g. increased cigarette smuggling, use of hand-rolled cigarettes [18,19,33].

It is more difficult to evaluate the effects of policies on risk perception. Italy has increased its health promotion interventions to prevent tobacco use after the WHO FCTC (World Health Assembly 2003), integrating institutional forces and professional figures outside of health education, helps to establish positive relationships with young people. Increased perception of smoking risk appears between 2000 and 2003, followed by a significant decrease, greater in males. The same prevalence between genders in the first period (Figure 2), suggests a similar external effect on perceived risk, but it is complicated to ascribe it to specific interventions programs, seems rather to be related to environmental background. Girls’ higher risk perception in the second period is in line with the literature suggesting their major susceptibility to policies directed to sensitize people on health consequences. In particular, attention to smoking health consequences further is evident in increased awareness of health risk through campaigns carried out since 2000, peaking in 2003 with law provided to report on cigarettes packs health warnings about smoking behaviour (Figure 1). Thus, this “warning” provided by law intervention, seems to have a short-term overall effect. Other studies indicate controversies of long-term beneficial effects of school or educational prevention programs. Similarly, the effect of health warnings on cigarettes packs on adolescent habits is also unclear; i.e. youth often ignored the messages [18,19,34,35]. These results may indicate limitations in efficacy of interventions directed to smoking risk, although among females seem to have a long-lasting effect.

Analysis performed with the two variables detecting trend before 2005 and period after, show similar trends across genders. In the first period we observe a consistent effect of policies: an increase of risk perception and a concomitant slowly decrease of perception of availability (Table 3). This may have effect on the decrease of experimenters (a decrease in lifetime use) but not on current smokers (not significant change in the last month use, note that “last month use” refers to use of cigarettes during the previous 30 days). Among last month smokers (Table 4), the increase of heavy smokers, the decrease of occasional smokers and the stability of moderate smokers suggest that some smokers changed their smoking habit: from occasional to moderate or heavy and from moderate to heavy. This may suggest that tobacco policies in the first period, had no effect regarding heavy smokers who probably had already established an addictive behaviour.

In the second period, with the absence of new policies (in particular laws) to increase awareness about risks associated with smoking, there is a decrease of risk perception. At the same time, probably the policies’ effect entered into force in 2005 and price policies, the perceived availability strongly decreases. Also in this period, risk and availability perception entail different effects on different smoker typologies: experimenters increase, whilst last month users are stable, there is an increase of moderate smokers, a decrease of occasional ones and a stability in the number of heavy smokers, that suggests a change of smoking habits of occasional smokers: from less than one cigarette per day to at least one per day. From the analysis above it is evident that risk and availability have different impacts based on variation in smokers typology.

Table 3 shows, in agreement with the literature, that “perceived risk but no availability” has a high “protective effect” on experimenters, but is stronger for the last month users [36]. This means that perceiving risk is protective against trying to smoke and, above all establishing a regular smoking behaviour. Once the adolescent becomes a smoker, to “perceive risk but no availability” has no effect in the conversion from occasional to moderate among males, but keeps its “protective” effect among females (Table 4). Among moderate smokers, males show a particular susceptibility only to availability. The attitude is different for heavy smokers: the odds to be a heavy smoker rather than an occasional one is greatly lower for youths that perceive risk but not availability. If a youth perceives smoking risk, he/she tends not to become a heavy smoker; if a youth doesn’t perceive risk but does perceive availability issues, he/she may become a heavy smoker, but perceiving both risk and availability has no effect.

Furthermore, there was a significant increase in odds in the addition of perceived availability, so that “not perceiving risk but perceiving availability” is a risk factor to try to smoke but also to become a smoker.

This may suggest that, in agreement with other studies, anti-smoking education should address each smoker typology differently [37,38], for example, for the heavy-smoker categories, establish a fundamental focus on the personal motivation and the process of intentional behavior change [39].

Unfortunately, “limit” refers to the term “availability” and is related to a self-perception; availability is multi-dimensional, and respondents may answer based on a wide range of factors, e.g. where to gain access, how to get there, and possibly cigarette cost.

This study has been able to determine the correlative relationship between perceived availability and risk and tobacco use but not the causal relationship [40].

The significance of our work results from access to large surveys of Italian high school students.

Finally, a consideration is that sampling school-based surveys does not capture those adolescents outside of the school environment: this may underestimate tobacco use across this age cohort [12].

## Conclusions

Tobacco use is variable among high-school students in Italy; in 2010 about 64% smoked at least one time and 7% are heavy smokers. A global decrease in lifetime and in last month use in recent years, is countered by stabilization in the number of heavy smokers.

It must be recognized that through the large number of legislative and social communication initiatives for the prevention of tobacco use in Italy, the situation has improved during the years in which such programs were active. In addition, it would be worth examining the influence of major social and cultural changes on smoking behaviors that were not addressed in the current survey because of their onset during the study period, itself, namely, the introduction and escalation of cell phone/text messaging/social networking as it has produced modification within the communication and support networks of adolescents in a significant manner.

The perception exists that smoking risk could prevent initial experimentation with tobacco among youths, but how such policies actually impact the perceived risk is not easily quantifiable. To understand the best strategies to implement will require additional studies that can collect data in support of separating the contributions of the individual programs.

The potential health risk associated with smoking habits and particularly with the established addiction at young age requires efforts focused at several levels. Effective measures need to be implemented targeting specific groups for direct action: 1) to prevent experimentation, it will be necessary to continually increase the risk perception among adolescents; 2) to decrease consumption among light smokers, interventions should focus on reducing availability; 3) to support smoking cessation, heavy-smokers should be targeted with comprehensive interventions and enforcement, identifying at risk adolescents, encouraging and assisting them with cessation; and 4) to promote “overall non-smoking”, change the approach to smoking within the community (e.g. promote smoke-free homes, parent-focused smoking prevention program, etc). Our results indicate different smoking attitudes depending on gender and age. This evidence increases our knowledge about the need for age-gender specific programs to produce more cost-effective measures.

In addition, we believe that this study provides an excellent example of the opportunity to expand the approaches and concepts of translational medicine and research to incorporate and effectively utilize observational studies to drive new clinical understanding and experimental design, with a focus on identifying critical clinical issues. While such studies do not operate in the conventional hypothesis-testing mode of more conventional clinical studies, it is clear that careful design, execution and analysis can lead to identification of new concepts that can readily lead to more conventional hypothesis-driven research, and become a significant component of the translational research process.

## Competing interests

All authors declare that they have no competing interests.

## Authors’ contributions

Participated in the conception and design of the study and the critical revision of the manuscript for important intellectual content: VS AP MG OC LF ML SM. Obtained funding for the study: SM. Performed the data analysis: VS AP MG SM. Coordinated the data collection and implemented the software for the data entry: LF. Had full access to all data (including statistical reports and tables) in the study and take responsibility for the integrity of the data and the accuracy of the data analysis: VS LF SM. Guarantor for the study: SM. Interpreted the data and produced the draft of the manuscript: VS AP MG OC ML SM. All authors read and approved the final manuscript.
